# Predictors and Impact of Arts Engagement During the COVID-19 Pandemic: Analyses of Data From 19,384 Adults in the COVID-19 Social Study

**DOI:** 10.3389/fpsyg.2021.626263

**Published:** 2021-04-26

**Authors:** Hei Wan Mak, Meg Fluharty, Daisy Fancourt

**Affiliations:** Department of Behavioural Science and Health, University College London, London, United Kingdom

**Keywords:** arts engagement, COVID-19, demographics, socio-economic position, psychosocial wellbeing, coping styles, emotion regulation

## Abstract

**Objectives:**

The global COVID-19 pandemic in 2020 heavily affected the arts and creative industries due to the instigation of lockdown measures in the United Kingdom and closure of venues. However, it also provided new opportunities for arts and cultural engagement through virtual activities and streamed performances. Yet it remains unclear (i) who was likely to engage with the arts at home during lockdown, (ii) how this engagement differed from patterns of arts engagement prior to COVID-19, and (iii) whether home-based arts engagement was related to people’s ability to cope with their emotions during lockdown. This study was therefore designed to address these questions.

**Methods:**

We used data collected in late May from the United Kingdom COVID-19 Social Study run by University College London. Multivariate regressions were used for the analysis (*N* = 19,384). Identified factors included demographic factors, socio-economic position, psychosocial wellbeing and health conditions, adverse events/worries, and coping styles.

**Results:**

Four types of home-based arts engagement were identified during the COVID-19 pandemic: digital arts and writing, musical activities, crafts, and reading for pleasure. Our results show that the strongest predictors of the engagement were age, education attainment, social support, and emotion-focused or supportive coping styles. In particular, younger adults (aged 18–29), non-keyworkers, people with greater social support, people who had lost work, those who were worried about catching the virus, and those with an emotion-focused, problem-focused or supportive coping style were more likely to have increased arts engagement during lockdown. Arts activities were used as approach and avoidance strategies to help cope with emotions, as well as to help improve self-development.

**Conclusion:**

Overall, our study suggests that while some people who engaged in the arts during the COVID-19 pandemic were those who typically engage under normal circumstances, the pandemic has also created new incentives and opportunities for others to engage virtually. Additionally, this study highlights the value of the arts as coping tools during stressful situations.

## Introduction

The global spread of COVID-19 in the early months of 2020 triggered monumental upheaval within the arts and creative industries. The instigation of lockdown measures in countries internationally led to the immediate closure of public spaces, galleries, exhibitions, museums, arts venues, and other cultural assets. A recent report from the Organisation for Economic Co-operation and Development OECD (2020) shows that the cultural sector was heavily affected by the pandemic due to a sharp drop in revenues and reductions of public and private funding for arts and culture. In the United Kingdom, for instance, enforced cancelations and closures in theaters led to over 15,000 canceled theatrical performances with a loss of over £303 million in box office revenue in the first 12 weeks of lockdown (House of Commons, 2020). While the United Kingdom government provided a rescue funding package for the cultural sector, the prolonged restrictions on social distancing still eventually led to many closures and redundancies in the sector (Arts Council England, 2020; House of Commons, 2020). However, the pandemic also provided new opportunities for arts engagement. Many arts and cultural providers began offering virtual activities to keep people engaged in digital arts activities (e.g., virtual museum tours), online groups (e.g., internet book clubs and virtual choirs), and streamed performances (e.g., concerts and plays). Further, many art forms became global lockdown trends, with viral videos of people singing from households and balconies around the world and a rapid increase in the sale of crafts materials such as paints and wools (Taylor, 2020). It has also been shown by a recent report from the United Kingdom Department for Digital, Culture, Media and Sport that, amongst over 1000 respondents, nearly half of them engaged in creative activities (e.g., story-writing, painting and drawing, designed video games) at home in May 2020 (DCMS, 2020). So whilst cultural engagement and community arts engagement were entirely ceased during strict lockdown, opportunities emerged for home-based arts engagement.

However, what remains unclear is who engaged with the arts at home in lockdown and how this engagement differed from patterns of arts engagement prior to COVID-19. Arts engagement has been found to be socially and geographically patterned (Mak et al., 2020a, b). Previous studies have shown that, in general, people who are younger, female, of white ethnicity, not married, and not living with children are more likely to participate in arts activities (including musical activities and visual and literature arts) (Devine and Dowds, 2013; Parkinson et al., 2014; Mak et al., 2020b). Further, it has been shown that individuals with higher educational qualifications and those in employment also have a higher engagement rate in the arts (Parkinson et al., 2014; Mak et al., 2020b). However, the sudden transition to solely home-based forms of arts engagement during the pandemic (especially the increase in online and digital arts engagement) might have changed the dynamics in the engagement. Borrowing the COM-B behavioral change model (Michie et al., 2011), the transition to new patterns of engagement might have attracted new participants who had lower engagement rates in usual times. The COM-B behavioral change model proposes that capabilities (i.e., knowledge and skills), opportunities (i.e., individuals’ social and physical environment), and motivations (i.e., reflective and automatic) are important factors for behavior change to occur (Michie et al., 2011). For instance, the wider access to arts and cultural programs and classes on the internet (e.g., online dance courses, sewing video tutorials) may have offered a chance for individuals to develop skills, creativity and confidence in the arts, building individual capability. Further, the proliferation of online opportunities may have provided opportunities to reach potential participants who might have been isolated from community activities or lived in areas with high levels of deprivation and few activities available, and have therefore missed out before the pandemic. Recent reports have also suggested that many individuals increased their arts engagement during this time due to a lack of opportunities to engage in many other leisure activities (Shehadi, 2020). In addition, the COVID-19 rules and regulations such as social distancing, travel restrictions, ‘stay at home’ orders and the closure of non-essential shops and entertainment may have motivated individuals to look for creative home-based activities including doing the arts. Indeed, a cross-country study in the United States and Korea found that moving arts and cultural activities online (e.g., virtual museums, arts galleries and live theaters) created more opportunities for children to experience the arts during COVID-19 (Choi et al., 2020). However, it is currently unclear how frequencies of arts engagement changed during COVID-19 compared to prior to the pandemic.

It is also unclear *who* engaged in the arts during the pandemic. There is a well-reported social gradient in arts engagement outside of pandemic circumstances (Parkinson et al., 2014; Mak et al., 2020b), and it is possible that this same social gradient was maintained during the pandemic. For instance, people from higher SES may have had greater resources and time to access to the arts virtually than those from lower SES who may have experienced economic challenges during the pandemic and hence have less time to spend on the arts (Choi et al., 2020). In addition, there is a recognized digital divide among people who have electronic devices and a good access to stable internet connection and those who do not have either (Choi et al., 2020). As a result, despite a wider access to arts and culture through the internet, the engagement rate may still be socially patterned. Moreover, for people who may previously have used the arts and cultural activities for social purposes, the sudden transition to solely home-based engagement may have reduced interest in engaging. As a consequence, it remains unknown whether the same social patterning of arts engagement reported in previous studies of audience demographics for the arts was maintained in lockdown during the pandemic, or whether new profiles of arts audiences emerged.

Another important question is whether this home-based arts engagement was associated with people’s abilities to cope during lockdown. Arts activities involve various components (e.g., imagination, sensory activation, cognitive stimulation and social interaction) that can prompt psychological, physiological, social and behavioral responses which are associated with the management of mental health and wellbeing (Fancourt and Finn, 2019). With an increasing number of people suffering from mental and emotional distress, depression, anxiety and loneliness during the pandemic (Bricker, 2020; Elran-Barak and Mozeikov, 2020; Groarke et al., 2020; Kwong et al., 2020; Pierce et al., 2020; Shanahan et al., 2020), arts and culture may have played a pivotal role in people’s mental health and wellbeing, such as through facilitating reduced stress levels, reduced risks of mental illness (e.g., depression and anxiety) and lower levels of loneliness through social interactions (Fancourt and Finn, 2019). This is supported by a recent study during COVID-19 that suggests that hobbies such as listening to music, reading and engaging in arts activates were associated with decreases in depressive symptoms and anxiety and increases in life satisfaction (Bu et al., 2020). Further, the arts have been shown to be effective at supporting individual’s coping, helping with the regulation of emotions through helping individuals to avoid stressors in their lives (e.g., by offering distraction), re-appraise problems they may be facing (e.g., through providing time and space to problem solve), and improve their self-confidence so they are better able to face challenges (Fancourt et al., 2019). This is particularly relevant given the arts and cultural activities could potentially help individuals to escape from negative emotions aroused by news related to COVID-19, as well as to reflect their emotions and derive a sense of accomplishment through skill-building in doing/learning the arts (Fancourt and Finn, 2019). However, there is currently little research on if and how home-based arts engagement during the pandemic helped in the regulation of emotions, and how this varied depending on type of arts activity and by personal characteristics.

Therefore, this paper explored three interconnected research questions (RQs):

1. What were the demographic, socio-economic and psychosocial predictors of home-based arts engagement during the first 10 weeks of strict lockdown in the United Kingdom?
2. How did frequency of arts engagement compare to prior to the pandemic amongst different subgroups?
3. How were different arts activities used to regulate emotions during lockdown amongst different subgroups?

To address the three RQs, we used statistical regression analysis to estimate the relationships between predictors and home-based arts engagement (RQ1), the frequency of the engagement (RQ2), and the use of arts for emotional regulation (RQ3). We hypothesized that the types of arts engagement, frequency of the engagement, and the use of arts for emotional regulation varied depending on people’s demographic background, socio-economic position, psychosocial wellbeing and health factors, adverse events and worries experienced during lockdown, and their personal coping styles.

## Materials and Methods

### Participants

This study analyzed data from the United Kingdom COVID-19 Social Study run by University College London, a longitudinal study that focuses on the psychological and social experiences of adults living in the United Kingdom during the COVID-19 pandemic. The study commenced on 21st March 2020 and involves weekly online data collection from participants for the duration of the pandemic. The study is not random and therefore is not representative of the United Kingdom population. However, it does contain a heterogeneous sample that was recruited using three primary approaches. First, snowballing was used, including promoting the study through existing networks and mailing lists (including large databases of adults who had previously consented to be involved in health research across the United Kingdom), print and digital media coverage, and social media. Second, more targeted recruitment was undertaken focusing on (i) individuals from a low-income background, (ii) individuals with no or few educational qualifications, and (iii) individuals who were unemployed. Third, the study was promoted via partnerships with third sector organizations to vulnerable groups, including adults with pre-existing mental health conditions, older adults, carers, and people experiencing domestic violence or abuse. The study was approved by the UCL Research Ethics Committee [12467/005] and all participants gave informed consent. A full protocol for the study is available online at www.COVIDSocialStudy.org.

Arts engagement was asked as a one-off module in week 10 of data collection from 21st May 2020 to 28th May 2020, with 28,743 participants completing the survey within these dates and providing responses to all measures on arts engagement. However, some participants opted not to provide details on their demographic backgrounds (e.g., gender and household income), so were excluded for these analyses, leaving a final sample with complete data of 19,384 participants.

### Measures

#### Arts Engagement

In week 10 of lockdown (21st May 2020), participants were asked in detail about their active arts engagement over the lockdown period (since 23rd March 2020). Questions explored 14 different types of arts engagement (either in person or virtually). A full list of variables is shown in Table 1. Responses measured on a five-point scale – “not at all,” “a few days,” “once or twice a week,” “most days,” and “every day.” Reponses to the original five-point scale are presented in Supplementary Table 1. These responses were collapsed into a binary indicator of “engaged” (those who reported engaging in any of these activities “a few days,” “once or twice a week,” “most days,” or “every day”) vs. “did not engage” (respondents who reported of not engaging in the activity at all), given that there was a large proportion of respondents reporting non-engagement in these activities (except for reading books where 33% reported “not at all” and listening to music where 19% reported “not at all”).

**TABLE 1:** Tetrachoric factor analysis for types of arts activities during the COVID-19 pandemic in the United Kingdom.

	Factor 1	Factor 2	Factor 3	Factor 4
	
	Digital arts and writing	Musical activities	Crafts	Reading for pleasure
Singing		0.8436		
Playing a musical instrument		0.4769		
Painting, drawing, printmaking or sculpture			0.5209	
Reading books, stories or poetry				0.8607
Textile crafts, e.g., embroidery, crocheting or knitting			0.6348	
Wood crafts, e.g., carving or furniture making			0.5674	
Other crafts, e.g., pottery, calligraphy or jewelry making			0.7979	
Creative writing	0.5895			
Dancing		0.7870		
Photography	0.6140			
Creating digital artworks or animations	0.7784			
Making films or videos	0.6988			
Listening to music		0.7127		
Other creative activity			0.5094	

*Factors loadings were produced by orthogonal rotation.*

Participants were also asked to rate whether their levels of arts engagement in April/May were less than usual (prior to the COVID-19 pandemic), about the same, or more than usual. In June/July, this question was repeated, this time asking people to compare their frequency of arts engagement in June/July (when the coronavirus restrictions were more relaxed) with the frequency in April/May.

#### Emotion Regulation Through Arts Engagement

To measure how respondents used artistic activities during the pandemic to regulate their emotions, we used the Emotion Regulation Strategies for Artistic Creative Activities Scale (ERS-ACA) (Fancourt et al., 2019). Respondents were given a set of 18 items (with a five-point scale ranging from “strongly disagree” to “strongly agree”) and were asked to what degree they agreed the statement when engaging in any of the arts activities. Three subscales were derived- “approach strategy” (six items such as acceptance and problem solving; alpha = 0.90), “avoidance strategy” (seven items such as distraction and detachment; alpha = 0.90), and “self-development strategy” (five items such as enhanced self-identity and improved self-esteem; alpha = 0.90). The approach and avoidance strategies have a correlation coefficient of 0.66; the approach and self-development strategies have a correlation coefficient of 0.77; and the avoidance and self-development strategies have a correlation coefficient of 0.69. A full list with factor loadings of the items is shown in Supplementary Table 2; the loadings were in line with those shown in the previous validation study (Fancourt et al., 2019).

#### Predictors/Covariates

In our analysis, we considered a rich set of demographic, socio-economic, psychosocial and health factors, adverse events and worries during lockdown, and coping styles as predictors of arts engagement. Demographic factors included respondents’ *age* (18–29 vs. 30–59 vs. 60+), *gender* (female vs. male), *ethnicity* (white ethnic vs. ethnic minority), *partnership status* (single and never married vs. divorced or widowed vs. in a relationship/married but living apart vs. in a relationship/married and cohabiting), *living arrangement* (living alone vs. not living alone and without children vs. not living alone and with children), and *living area* (living in city/town vs. living in remote suburban areas).

Socio-economic factors included *employment status* (full-time employment/self-employed vs. part-time employment vs. economically inactive [e.g., student/retired/homemakers/unable to work due to disability] vs. unemployed and seeking work), *educational levels* (undergraduate degree/professional qualification/postgraduate degree vs. post-16 vocational course/A-levels [subject specific qualifications typically taken at age 18] or equivalent [at school until age 18] vs. completed GCSE/CSE/O-levels [subject specific qualifications typically taken at age 16] or equivalent [at school until age 16]/no qualifications), *household income* (>£30,000 vs. <£30,000 total household income per annum), *housing space* (overcrowded household [defined as more than one person per room in the house, excluding bathrooms and kitchen] vs. not overcrowded), whether respondents were *keyworkers* and whether respondents were *house owners*.

We also controlled for three psychosocial wellbeing measures and two health conditions. The three psychosocial wellbeing measures include *social support*, an adapted version of the 6-item short form of Perceived Social Support Questionnaire (F-SozU K-6). Each item is rated on a five-point scale from “not true at all” to “very true,” with higher scores indicating higher levels of perceived social support. Minor adaptations were made to the language in the scale to make it relevant to experiences during COVID-19 (Supplementary Table 3) (Kliem et al., 2015; Lin et al., 2019); *size of social network* (large network with ≥3 friends vs. small network with <3 friends); *loneliness*, which was using the 3-item UCLA-3 loneliness [a short form of the Revised UCLA Loneliness Scale (UCLA-R)], with an additional item asking how often respondents felt lonely. Each item is rated with a three-point rating scale, ranging from “hardly ever” to “often,” with higher scores indicating greater loneliness (Russell et al., 1980). The two health conditions include whether respondents had any of the following diagnosed *mental health conditions:* clinically diagnosed anxiety, clinically diagnosed depression, or other clinically diagnosed mental health problem; and whether they had any of the following diagnosed *physical condition or disability:* high blood pressure, diabetes, heart disease, lung disease, cancer, another clinically diagnosed chronic physical health condition, a disability that affects ability to leave the house, or another disability.

We also considered respondents’ adverse events and worries experienced during the pandemic. For adverse events, these include *COVID-19 diagnosis* (diagnosed and recovered or diagnosed and still ill or not formally diagnosed but suspected vs. no diagnosis), *physical/psychological abuse* (being physically harmed/hurt by somebody else or being bullied, controlled, intimidated or psychologically hurt by someone else vs. no abuse), *financial difficulties* (unable to pay bills/rent/mortgage or had a major cut in household income vs. no difficulties), *lost work* (lost their job/unable to do paid work vs. did not lose work), *difficulties accessing food* (unable to access sufficient food vs. no difficulties) and *difficulties accessing medication* (unable to access required medication vs. no difficulties). For worries, these include individuals indicating whether of the following items were a source of minor and/or major stress (defined as stress that was constantly on their mind or kept them awake): *COVID-19 stress* (worried about catching COVID-19 or becoming serious ill from COVID-19), *worries over personal safety*, *worries over finances*, *worries over unemployment, worries over food access*, and *worries over medication access*.

Lastly, our model controlled for respondents’ coping styles. Coping is broadly defined as the cognitive and behavioral efforts and individuals employ to manage stress (Ray and Gibson, 1982; Lazarus and Folkman, 1991). These behaviors are often referred to as strategies, and may be either conscious or unconscious (Lazarus and Folkman, 1991). There are a number of ways to categorize coping strategies, which largely center around stressor and one’s actions (or inactions) toward it (Aspinwall and Taylor, 1997). We measured coping using the 28-item Brief COPE scale (Carver, 1997) and in line with previous research, we used a four-factor model for our analysis: *problem-focused coping style, emotion-focused coping style, avoidant coping style*, and *supportive coping style* (Nahlen Bose et al., 2015).

### Analyses

To identify the underlying latent categories of arts engagement, we ran a factor analysis of the matrix of tetrachoric correlations using all the arts activity measures. The Kaiser–Meyer–Olkin measure of sampling adequacy was 82.4 (meritorious). The Kaiser’s criterion of eigenvalues >1 clearly indicated a four-factor structure, and inspection of a scree plot confirmed this was a reasonable choice (Kaiser, 1960). The four-factor loading was the same in oblique and orthogonal rotations.

Four arts activities were loaded on Factor 1 (labeled as “digital arts and writing”), which included creative writing, photography, creating digital artworks or animations, and making films or videos. Factor 2 (labeled as “musical activities”) had four factor loadings including singing, playing a musical instrument, dancing, and listening to music. Factor 3 (labeled as “crafts”) was comprised of five loadings, including painting, drawing, printmaking or sculpture, textile crafts (e.g., embroidery, crocheting or knitting), wood crafts (e.g., carving or furniture making), other crafts (e.g., pottery, calligraphy or jewelry making) and other creative activity. Finally, factor 4 (labeled as “reading for pleasure”) only had one loading. We generated a binary indicator for whether respondents had engaged in any activity within each of the four categories during lockdown (Table 1).

Given the various nature of the outcome variables (binary, categorical, and continuous variables), different forms of regression analysis were applied in this study:

For RQ1, multivariate logistic regression was applied to calculate the odds ratio (OR) and 95% confidence intervals (CIs) to predict how likely participants were to have engaged in each of the arts engagement behavior based on predictors. Five sets of models were performed by entering different sets of covariates sequentially. Model 1 examined the relationship between demographic factors and arts engagement. Model 2 additionally included socio-economic position to Model 1. Model 3 additionally adjusted for psychosocial wellbeing and health conditions. Model 4 additionally controlled for adverse events/worries, and finally, Model 5 additionally considered coping styles in the model.

For RQ2, we used multinomial logistic regression to estimate the relative risk ratio (RRR) of whether people had been engaging more or less arts activities than usual. Similar to RQ1, model was sequentially adjusted for all covariates.

For RQ3, ordinary least squares (OLS) regression was applied to identify the predictors of the emotional regulation strategies through arts engagement. Coefficients and 95% CIs were provided to indicate the direction of the relationship between a predictor and each of the emotion regulation strategies. Model was adjusted for all covariates.

To balance the data against population demographics, we weighted data to match the core demographic features of the target population (namely gender, age groups, ethnicity, education and country of living including England, Wales, Scotland, and Northern Ireland) obtained from the Office for National Statistics (2020). The Stata user-written command ‘ebalance’ were used for weighting for the selected analytical sample. Full details on study sample, procedures and content are provided in the online user guide^1^. As multiple regression models were applied in this study, we adjusted the p-value to 0.01 to produce more conservative results. All analyses were carried out in Stata v16.1.

## Results

In our weighted sample, 10% were aged 18–29, 47% aged 30–59 and 43% aged 60 or above. Half of the sample were female, 92% were of white ethnic, and 22% of the sample were living alone. On average, 38% had a degree or above, 52% had an annual household income of >£30,000, and 41% of the sample were in full-time employment or were self-employed, whilst 45% were economically inactive (e.g., students and the retired) (Table 2).

**TABLE 2:** Descriptive statistics of the analytical sample (unweighted and weighted).

	Mean (SE)/%	
	Unweighted; *N* = 19,673	Weighted; *N* = 19,384
**Demographic backgrounds**		
Ages 18–29	6.03	9.53
Ages 30–59	57.0	47.0
Ages 60+	37.0	43.4
Female vs. male	75.2 vs. 24.8	50.7 vs. 49.3
White ethnic vs. ethnic minority	96.4 vs. 3.60	92.2 vs. 7.79
Single and never married	15.6	16.7
Divorced or widowed	14.6	14.9
In a relationship/married but living apart	5.62	5.94
In a relationship/married and cohabiting	64.2	62.2
Living alone	21.8	21.9
Not living alone and without children	55.8	58.4
Not living alone and with children	22.4	19.7
Living in city/town vs. living in village/hamlet/isolated dwelling	75.6 vs. 24.4	77.1 vs. 22.9
**Socio-economic position**		
Full-time employment/self-employed	45.6	41.3
Part-time employment	15.2	11.7
Economically inactive (incl. student/retired/homemakers/unable to work due to disability)	37.5	44.8
Unemployed and seeking work	1.76	2.14
GCSE/CSE/O-levels or equivalent or below	12.6	29.8
Post-16 vocational or A-levels qualifications or equivalent	16.8	32.5
Degree or above	70.6	37.8
Household income >£30,000 vs. household income <£30,000	60.7 vs. 39.3	51.6 vs. 48.4
Not living in overcrowded households vs. living in overcrowded households	91.8 vs. 8.17	89.8 vs. 10.2
Keyworkers vs. not non-keyworkers	21.1 vs. 79.0	18.7 vs. 81.3
House owners vs. not house owners	75.4 vs. 24.6	69.5 vs. 30.5
**Psychosocial measures and health conditions**		
Social support (ranging from 6 to 30)	22.6 (0.05)	22.0 (0.08)
Social network (≥3 friends vs. <3 friends)	75.3 vs. 24.7	70.3 vs. 29.7
Loneliness (ranging from 4 to 12)	6.33 (0.02)	6.33 (0.03)
Diagnosed mental health condition vs. no condition	16.8 vs. 83.2	16.8 vs. 83.2
Diagnosed physical health condition or disability^1^ vs. no condition	41.1 vs. 58.9	45.3 vs. 54.7
**Adverse events**		
COVID-19 diagnosis vs. no COVID-19 diagnosis	12.1 vs. 87.9	10.9 vs. 89.1
Physically/psychologically abused vs. not abused	4.72 vs. 95.3	4.71 vs. 95.3
Financial difficulties vs. no difficulties	10.3 vs. 89.7	10.9 vs. 89.1
Lost work vs. did not lose work	4.35 vs. 95.7	4.43 vs. 95.6
Difficulties accessing food vs. no difficulties	1.25 vs. 98.8	1.67 vs. 98.3
Difficulties accessing medication vs. no difficulties	1.56 vs. 98.4	1.83 vs. 98.2
**Worries**		
Catching COVID-19 vs. not worried	42.7 vs. 57.4	41.8 vs. 58.3
Personal safety vs. not worried	9.47 vs. 90.5	9.72 vs. 90.3
Finances vs. not worried	25.5 vs. 74.5	26.6 vs. 73.4
Unemployment vs. not worried	12.6 vs. 87.4	12.2 vs. 87.8
Food access vs. not worried	7.67 vs. 92.3	7.59 vs. 92.4
Medication access vs. not worried	5.69 vs. 94.3	5.71 vs. 94.3
**Coping styles**		
Problem-focused coping	0.09 (0.00)	0.01 (0.01)
Emotion-focused coping	0.11 (0.00)	0.01 (0.01)
Avoidant coping	0.00 (0.00)	−0.01 (0.01)
Supportive coping	0.13 (0.00)	−0.01 (0.01)
**Arts activities**		
Digital arts and writing vs. did not do any digital arts or writing	32.7 vs. 67.3	28.9 vs. 71.1
Musical activities vs. did not do any musical activities	84.8 vs. 15.2	83.9 vs. 16.1
Crafts vs. did not engage do any crafts	49.5 vs. 50.5	42.0 vs. 58.0
Reading for pleasure vs. did not read for pleasure	75.9 vs. 24.1	67.4 vs. 32.6
**Emotion Regulation Strategies for Artistic Creative Activities (ERS-ACA)^2^**		
Approach (ranging from 6 to 30)	19.0 (0.03) (*N* = 18,831)	18.9 (0.05) (*N* = 18,564)
Avoidance (ranging from 7 to 35)	24.1 (0.04) (*N* = 18,831)	23.6 (0.06) (*N* = 18,564)
Self-development (ranging from 5 to 25)	16.0 (0.03) (*N* = 18,831)	15.7 (0.05) (*N* = 18,564)

*Data were weighted to the proportion of gender, age, ethnicity, education and country of living (i.e., England, Wales, Scotland, and Northern Ireland) obtained from the Office for National Statistics (2020).*

*^1^The prevalence of physical health conditions reported here is in line with a National Health Service report (2018): https://www.england.nhs.uk/blog/making-the-case-for-the-personalised-approach.*

*^2^A reduction in sample here is due to the condition of restricting the sample to those with complete responses to the set of ERS-ACA items for direct comparisons. This condition was not applied in regression analyses, where a score was generated for every respondent for which there was a response to one or more items. The summative score was averaged and then standardized.*

### RQ1: Predictors of Arts Engagement

#### Demographic Backgrounds

Younger adults (aged 18–29) were more likely to engage in all kinds of arts activities apart from reading for pleasure during the pandemic, whereas older adults (aged 60+) were more likely to do crafts (OR = 1.15) and read for pleasure (OR = 1.72) but were less likely to engage in musical activities (OR = 0.56), compared to adults aged 30–59. Females had a 2.2 to 2.3 times higher odds of reading and doing crafts, respectively. In comparison to those who were single and never married, respondents who were divorced or widowed were less likely to engage in digital arts and writing (OR = 0.75), whereas people who were living with a partner were more likely to read for pleasure (OR = 1.27). However, respondents who lived with children had a 26% lower odds of reading. Higher engagement in digital arts and writing (OR = 1.28) was found in people living in remote suburban areas. No associations were found between ethnicity and home-based arts activities (Table 3).

**TABLE 3:** Logistic regression predicting the types of arts activities during the COVID-19 pandemic in the United Kingdom (weighted; *N* = 19,384).

	Digital arts and writing			Musical activities			Crafts			Reading for pleasure		
				
	OR	95% CI	*P*-value	OR	95% CI	*P*-value	OR	95% CI	*P*-value	OR	95% CI	*P*-value
**Model 1: Demographic backgrounds**												
Ages 18–29	1.54	1.27–1.86	0.000	1.88	1.34–2.65	0.000	1.49	1.24–1.79	0.000	1.30	1.05–1.61	0.018
Ages 60+	0.94	0.84–1.05	0.252	0.56	0.49–0.65	0.000	1.15	1.04–1.27	0.007	1.72	1.52–1.93	0.000
(ref: ages 30–59)												
Female	1.02	0.92–1.12	0.740	0.87	0.77–0.98	0.024	2.27	2.07–2.49	0.000	2.24	2.03–2.48	0.000
(ref: male)												
White ethnic	0.95	0.75–1.20	0.656	0.76	0.55–1.06	0.107	1.27	1.03–1.57	0.029	1.36	1.08–1.72	0.010
(ref: ethnic minority)												
Divorced or widowed	0.75	0.63–0.90	0.002	1.03	0.83–1.29	0.772	0.83	0.70–0.97	0.021	0.86	0.71–1.03	0.103
In a relationship/married but living apart	0.90	0.71–1.14	0.395	1.42	1.02–1.96	0.037	1.04	0.83–1.30	0.721	1.01	0.79–1.29	0.953
In a relationship/married and cohabiting	0.84	0.71–0.99	0.042	1.11	0.88–1.41	0.369	1.04	0.89–1.22	0.601	1.27	1.07–1.52	0.008
(ref: single and never married)												
Not living alone and without children	1.06	0.89–1.25	0.524	0.90	0.73–1.11	0.324	1.14	0.98–1.34	0.090	0.93	0.78–1.10	0.388
Not living alone and with children	0.96	0.79–1.16	0.687	0.82	0.63–1.07	0.149	1.06	0.88–1.26	0.549	0.74	0.61–0.90	0.003
(ref: living alone)												
Living in village/hamlet/isolated dwelling	1.28	1.15–1.42	0.000	0.90	0.79–1.03	0.125	1.08	0.97–1.20	0.144	0.99	0.88–1.11	0.875
(ref: living in city/town)												
Constant	0.45	0.34–0.59	0.000	9.44	6.55–13.61	0.000	0.31	0.24–0.40	0.000	0.81	0.62–1.06	0.126
**Model 2: Model 1 + socio-economic position**												
Full-time employment/self-employed	0.81	0.58–1.14	0.223	1.23	0.77–1.97	0.379	0.85	0.62–1.18	0.342	0.87	0.61–1.24	0.440
Part-time employment	0.81	0.57–1.15	0.247	1.10	0.67–1.80	0.700	0.92	0.66–1.29	0.635	1.11	0.76–1.61	0.594
Economically inactive (incl. student/retired/homemakers/unable to work due to disability)	0.77	0.55–1.09	0.137	0.93	0.58–1.50	0.773	0.90	0.65–1.25	0.528	0.98	0.69–1.41	0.931
(ref: unemployed and seeking work)												
Post-16 vocational or A-levels qualifications or equivalent	1.81	1.54–2.12	0.000	1.28	1.09–1.51	0.003	1.19	1.05–1.36	0.009	1.59	1.38–1.83	0.000
Degree or above	2.47	2.14–2.85	0.000	1.48	1.29–1.70	0.000	1.62	1.45–1.83	0.000	2.99	2.63–3.40	0.000
(ref: GCSE/CSE/O-levels or equivalent or below)												
Household income >£30,000	1.04	0.92–1.18	0.501	1.05	0.91–1.21	0.513	0.85	0.76–0.95	0.004	1.12	0.99–1.27	0.065
(ref: household income <£30,000)												
Not living in overcrowded households	0.94	0.78–1.13	0.509	1.19	0.94–1.51	0.140	1.01	0.85–1.20	0.885	1.10	0.91–1.33	0.310
(ref: living in overcrowded households)												
Non-keyworkers	1.45	1.27–1.64	0.000	1.01	0.85–1.20	0.885	1.28	1.14–1.44	0.000	1.31	1.15–1.50	0.000
(ref: keyworkers)												
House owners	0.80	0.71–0.91	0.001	0.96	0.82–1.11	0.571	0.93	0.83–1.04	0.220	1.21	1.07–1.37	0.003
(ref: not house owners)												
Constant	0.23	0.15–0.38	0.000	5.30	2.81–10.01	0.000	0.23	0.15–0.36	0.000	0.28	0.17–0.46	0.000
**Model 3: Model 2 + psychosocial wellbeing and health conditions**												
Social support	1.02	1.02–1.03	0.000	1.04	1.03–1.05	0.000	1.03	1.02–1.04	0.000	1.02	1.01–1.03	0.000
Large social network (≥3 friends)	1.14	1.02–1.29	0.026	1.33	1.16–1.52	0.000	1.13	1.02–1.26	0.021	1.30	1.15–1.46	0.000
Loneliness	1.04	1.01–1.07	0.005	1.03	0.99–1.06	0.113	1.01	0.99–1.03	0.460	0.97	0.94–0.99	0.015
Diagnosed mental health condition	1.06	0.92–1.21	0.428	1.03	0.88–1.22	0.702	1.25	1.10–1.41	0.000	0.96	0.83–1.10	0.567
Diagnosed physical health condition or disability	0.98	0.88–1.08	0.627	0.85	0.75–0.97	0.013	0.96	0.88–1.06	0.412	0.97	0.87–1.08	0.526
Constant	0.12	0.07–0.21	0.000	2.72	1.33–5.58	0.006	0.14	0.08–0.23	0.000	0.27	0.16–0.48	0.000
**Model 4: Model 3 + adverse events/worries**												
Adverse events												
COVID-19 diagnosis	1.18	1.01–1.36	0.031	1.04	0.85–1.28	0.681	1.09	0.95–1.25	0.239	1.15	0.99–1.35	0.076
Physically/psychologically abused	1.23	0.97–1.56	0.090	0.84	0.64–1.11	0.218	0.93	0.75–1.15	0.493	0.90	0.71–1.15	0.407
Financial difficulties	1.03	0.86–1.22	0.769	0.94	0.74–1.19	0.614	1.15	0.98–1.35	0.096	0.90	0.76–1.08	0.267
Lost work	1.57	1.24–1.99	0.000	1.23	0.87–1.74	0.251	1.23	0.96–1.56	0.097	1.10	0.84–1.45	0.485
Difficulties accessing food	1.00	0.59–1.71	0.989	1.07	0.63–1.83	0.792	0.78	0.52–1.19	0.249	1.09	0.69–1.72	0.710
Difficulties accessing medication	0.82	0.51–1.30	0.394	1.15	0.67–1.98	0.607	1.14	0.75–1.73	0.550	0.88	0.57–1.35	0.554
Worries												
Catching COVID-19	1.18	1.07–1.31	0.001	1.20	1.05–1.37	0.006	1.11	1.01–1.22	0.034	0.93	0.83–1.04	0.188
Personal safety	1.25	1.06–1.47	0.009	0.98	0.79–1.23	0.890	0.99	0.85–1.16	0.936	1.02	0.85–1.23	0.809
Finances	1.04	0.92–1.19	0.502	1.08	0.90–1.28	0.410	1.16	1.02–1.30	0.019	0.94	0.82–1.07	0.342
Unemployment	1.12	0.96–1.31	0.165	0.99	0.79–1.24	0.939	0.99	0.85–1.15	0.874	1.09	0.92–1.30	0.311
Food access	1.00	0.81–1.23	0.987	0.82	0.65–1.05	0.111	1.08	0.89–1.30	0.429	0.90	0.73–1.11	0.343
Medication access	1.29	1.01–1.66	0.044	0.93	0.70–1.23	0.596	1.13	0.90–1.41	0.292	1.05	0.81–1.36	0.715
Constant	0.10	0.06–0.18	0.000	2.65	1.28–5.47	0.009	0.13	0.07–0.21	0.000	0.27	0.15–0.49	0.000
**Model 5: Model 4 + coping styles**												
Problem-focused coping	1.31	1.15–1.49	0.000	1.21	1.02–1.42	0.025	1.48	1.32–1.66	0.000	1.13	0.98–1.30	0.081
Emotion-focused coping	1.29	1.18–1.41	0.000	1.26	1.12–1.43	0.000	1.17	1.08–1.27	0.000	1.24	1.12–1.37	0.000
Avoidant coping	0.90	0.81–1.00	0.055	0.93	0.81–1.06	0.257	0.94	0.85–1.03	0.191	0.91	0.82–1.02	0.108
Supportive coping	1.27	1.16–1.39	0.000	1.33	1.17–1.50	0.000	1.12	1.03–1.22	0.007	1.31	1.19–1.45	0.000
Constant	0.18	0.10–0.32	0.000	4.97	2.32–10.64	0.000	0.19	0.11–0.32	0.000	0.47	0.26–0.84	0.011

#### Socio-Economic Factors

Whilst employment status was not associated with any arts activities, people with higher education levels were more likely to engage in all kinds of activities. In particular, those with a degree or above qualification had a 1.5 to 3 times higher odds of engaging in the arts. People with an annual household income of >£30k had a 15% lower odds of engaging in crafts activities. While living space was not related to any of the arts activities, respondents who were a house owner had a 20% lower odds of engaging in digital arts and writing but had a 21% higher odds of reading for pleasure. Higher engagement in digital arts and writing, crafts activities and reading was also found in individuals who were not keyworkers (Table 3).

#### Psychosocial and Health Factors

People with higher levels of perceived social support were more likely to engage in all kinds of art activities. Respondents with a larger social network had a 33% higher odds of doing musical activities and a 30% higher odds of reading for pleasure. Whilst people who felt lonelier were more likely to engage in digital arts and writing (OR = 1.04), those with a diagnosed mental health condition had a 25% higher odds of doing crafts. No associations were found between physical health diagnosis and home-based arts activities (Table 3).

#### Adverse Events/Worries

Respondents who lost their job during the pandemic had a 57% higher odds in doing digital arts and writing. No associations were found for other adverse events. Individuals who were worried about catching COVID-19 were more likely to engage in digital arts and writing (OR = 1.18) and musical activities (OR = 1.20). Those who were worried about their personal safety had a 25% higher odds of engaging in digital arts and writing. No associations were found for other worries (Table 3).

#### Coping Styles

Respondents with emotion-focused and supportive coping styles were more likely to engage in all kinds of arts activities, while those with a problem-focused coping style were more likely to engage in digital arts and writing (OR = 1.31) and crafts activities (OR = 1.48). No associations were found between an avoidant coping style and home-based arts activities (Table 3).

#### Sensitivity Analysis

In addition to ORs, we have also provided marginal effects to present results as differences in probabilities, which can help provide a sense of the magnitude (Supplementary Table 4). For example, we found that whilst females had a 17% and 19% higher probability in reading and crafts activities than males, respectively, younger adults had a 6–9% higher probability in engaging all kinds of activities than adults aged 30–59. Particularly, our study shows that, compared to those with a qualification up to GCSE or equivalent, respondents with a degree or above qualification had a 17%, 5%, 11%, and 22% higher probability in doing digital arts and writing, musical activities, crafts activities and reading for pleasure, respectively. Given that “listening to music” is a ubiquitous activity, we conducted a sensitivity analysis for musical activities by omitting this activity. When focusing on activities like singing, playing a musical instrument, and dancing, we found that younger adults (aged 18–29), female, people of ethnic minority, those who were single and never married, people who lived with children, those with higher education level, people with greater levels of perceived social support, those who had been diagnosed COVID-19, and people with emotional-focused or supportive coping styles engaged more in these activities (Supplementary Table 5).

### RQ2: Frequency of Arts Engagement

**FIGURE 1:**
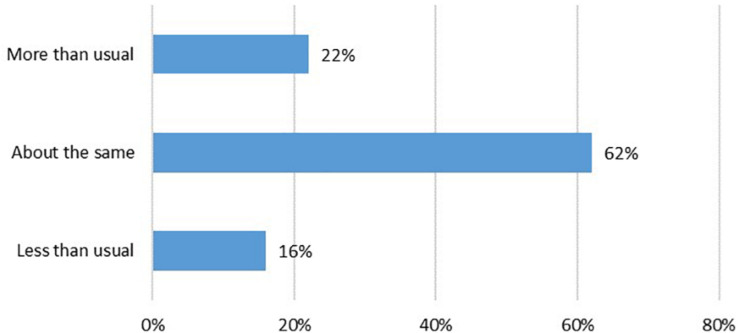
Frequency of arts engagement in April/May during lockdown vs. pre-pandemic.

In our sample, 16% of people reported that they had decreased their participation in the arts during the first lockdown in April/May compared to prior to the pandemic, 62% had about the same amount of engagement levels before and during the pandemic, and 22% increased their engagement (Figure 1).

**FIGURE 2:**
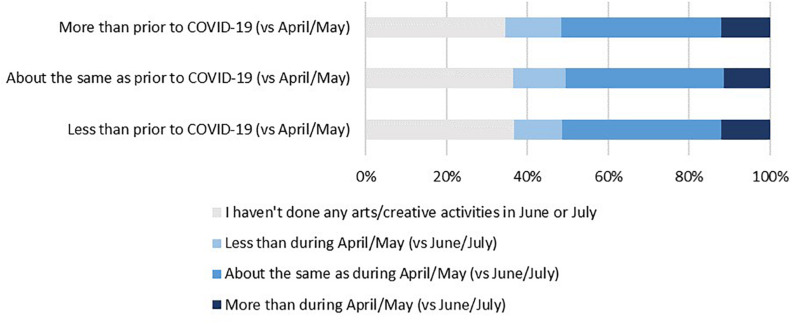
Frequency of arts engagement in April/May during lockdown vs. engagement across June/July.

When re-measuring in June/July where lockdown restrictions had eased, 52% of respondents who reported increasing their arts engagement during lockdown had either remained high levels of engagement or had further increased their engagement 3 months later. Conversely, 51% of respondents reported that their arts engagement decreased during lockdown had either remained low engagement or had further lowered 3 months later (Figure 2).

When comparing the amount of arts engagement during the COVID-19 pandemic to the amount during usual times (i.e., prior to the pandemic), groups who increased their arts engagement included adults who were younger (aged 18–29), non-keyworkers, those with greater social support, people who had lost work, those who were worried about catching COVID-19, and those who had an emotion-focused, problem-focused or supportive coping style. In contrast, older adults (aged 60+) and individual who were economically inactive were less likely to have increased their engagement (Table 4).

**TABLE 4:** Multinomial logistic regression predicting the amount of arts engagement in April/May during the COVID-19 pandemic compared to usual amount (prior to the pandemic) (weighted; *N* = 19,384).

	Less than usual vs. about the same as usual			More than usual vs. about the same as usual		
		
	RRR	95% CI	*P*-value	RRR	95% CI	*P*-value
**Model 1: Demographic backgrounds**						
Ages 18–29	1.14	0.86–1.52	0.363	1.44	1.19–1.76	0.000
Ages 60+	1.15	1.00–1.32	0.053	0.66	0.58–0.76	0.000
(ref: ages 30–59)						
Female	1.57	1.38–1.78	0.000	2.19	1.95–2.46	0.000
(ref: male)						
White ethnic	0.77	0.58–1.03	0.076	1.01	0.79–1.31	0.915
(ref: ethnic minority)						
Divorced or widowed	0.97	0.78–1.21	0.804	0.84	0.69–1.03	0.089
In a relationship/married but living apart	1.18	0.87–1.61	0.294	1.02	0.80–1.31	0.863
In a relationship/married and cohabiting	0.91	0.73–1.14	0.417	1.02	0.85–1.24	0.801
(ref: single and never married)						
Not living alone and without children	0.88	0.71–1.08	0.232	0.99	0.83–1.19	0.953
Not living alone and with children	0.81	0.64–1.03	0.090	1.00	0.81–1.24	0.980
(ref: living alone)						
Living in village/hamlet/isolated dwelling	0.86	0.75–0.99	0.041	0.88	0.79–1.00	0.041
(ref: living in city/town)						
Constant	0.29	0.21–0.41	0.000	0.27	0.20–0.36	0.000
**Model 2: Model 1 + socio-economic position**						
Full-time employment/self-employed	0.61	0.39–0.94	0.025	0.77	0.53–1.12	0.174
Part-time employment	0.62	0.39–0.98	0.040	0.81	0.55–1.19	0.291
Economically inactive (incl. student/retired/homemakers/unable to work due to disability)	0.84	0.54–1.31	0.451	0.54	0.37–0.79	0.002
(ref: unemployed and seeking work)						
Post-16 vocational or A-levels qualifications or equivalent	1.30	1.08–1.56	0.006	1.22	1.04–1.44	0.018
Degree or above	1.64	1.39–1.94	0.000	1.52	1.32–1.76	0.000
(ref: GCSE/CSE/O-levels or equivalent or below)						
Household income >£30,000	1.07	0.91–1.25	0.419	1.04	0.91–1.18	0.573
(ref: household income <£30,000)						
Not living in overcrowded households	0.89	0.71–1.12	0.307	1.06	0.87–1.29	0.577
(ref: living in overcrowded households)						
Non-keyworkers	1.17	0.97–1.42	0.110	1.31	1.15–1.49	0.000
(ref: keyworkers)						
House owners	0.91	0.77–1.07	0.236	0.96	0.84–1.09	0.508
(ref: not house owners)						
Constant	0.31	0.17–0.55	0.000	0.21	0.13–0.35	0.000
**Model 3: Model 2 + psychosocial wellbeing and health conditions**						
Social support	0.99	0.98–1.00	0.083	1.02	1.01–1.03	0.000
Large social network (≥3 friends)	1.32	1.13–1.55	0.001	1.34	1.18–1.52	0.000
Loneliness	1.22	1.18–1.25	0.000	1.06	1.03–1.09	0.000
Diagnosed mental health condition	1.26	1.06–1.49	0.009	1.22	1.05–1.41	0.008
Diagnosed physical health condition or disability	1.13	0.99–1.30	0.069	0.92	0.83–1.03	0.152
Constant	0.05	0.02–0.10	0.000	0.08	0.05–0.15	0.000
**Model 4: Model 3 + adverse events/worries**						
Adverse events						
COVID-19 diagnosis	0.97	0.81–1.17	0.756	1.08	0.92–1.26	0.356
Physically/psychologically abused	1.76	1.34–2.30	0.000	1.07	0.79–1.45	0.651
Financial difficulties	1.15	0.92–1.43	0.211	1.14	0.94–1.38	0.188
Lost work	1.53	1.10–2.12	0.011	1.47	1.11–1.95	0.008
Difficulties accessing food	1.25	0.74–2.09	0.400	1.37	0.79–2.37	0.259
Difficulties accessing medication	1.92	1.16–3.18	0.011	2.04	1.18–3.51	0.011
Worries						
Catching COVID-19	1.19	1.04–1.36	0.012	1.21	1.09–1.35	0.001
Personal safety	1.41	1.14–1.75	0.002	1.13	0.92–1.38	0.259
Finances	1.15	0.97–1.36	0.113	1.04	0.90–1.19	0.602
Unemployment	1.14	0.92–1.40	0.233	1.08	0.91–1.28	0.366
Food access	0.89	0.70–1.14	0.370	0.93	0.73–1.19	0.581
Medication access	1.14	0.84–1.53	0.405	0.93	0.69–1.25	0.632
Constant	0.04	0.02–0.08	0.000	0.07	0.04–0.13	0.000
**Model 5: Model 4 + coping styles**						
Problem-focused coping	1.19	1.01–1.40	0.036	1.24	1.09–1.42	0.001
Emotion-focused coping	1.06	0.94–1.19	0.340	1.35	1.23–1.48	0.000
Avoidant coping	1.27	1.10–1.45	0.001	0.89	0.79–1.00	0.058
Supportive coping	1.19	1.04–1.35	0.010	1.14	1.03–1.25	0.009
Constant	0.06	0.03–0.13	0.000	0.10	0.06–0.19	0.000

Conversely, groups who had decreased their arts engagement included people with post-16 vocational/A-levels qualifications or equivalent, those who had been physically or psychologically abused, people who were worried about their personal safety and those with an avoidant coping style (Table 4).

There were additional factors that indicated a change in the amount of arts engagement, but operated in both directions, indicating a greater likelihood of changing patterns amongst these groups but less consistency in the direction of that change. These included female gender, people with a degree or above education level, those with a larger social network, people with higher levels of loneliness, and those with a diagnosed mental health condition (Table 4).

### RQ3: Use of Arts to Regulate Emotions

#### Type of Arts Activities

**FIGURE 3:**
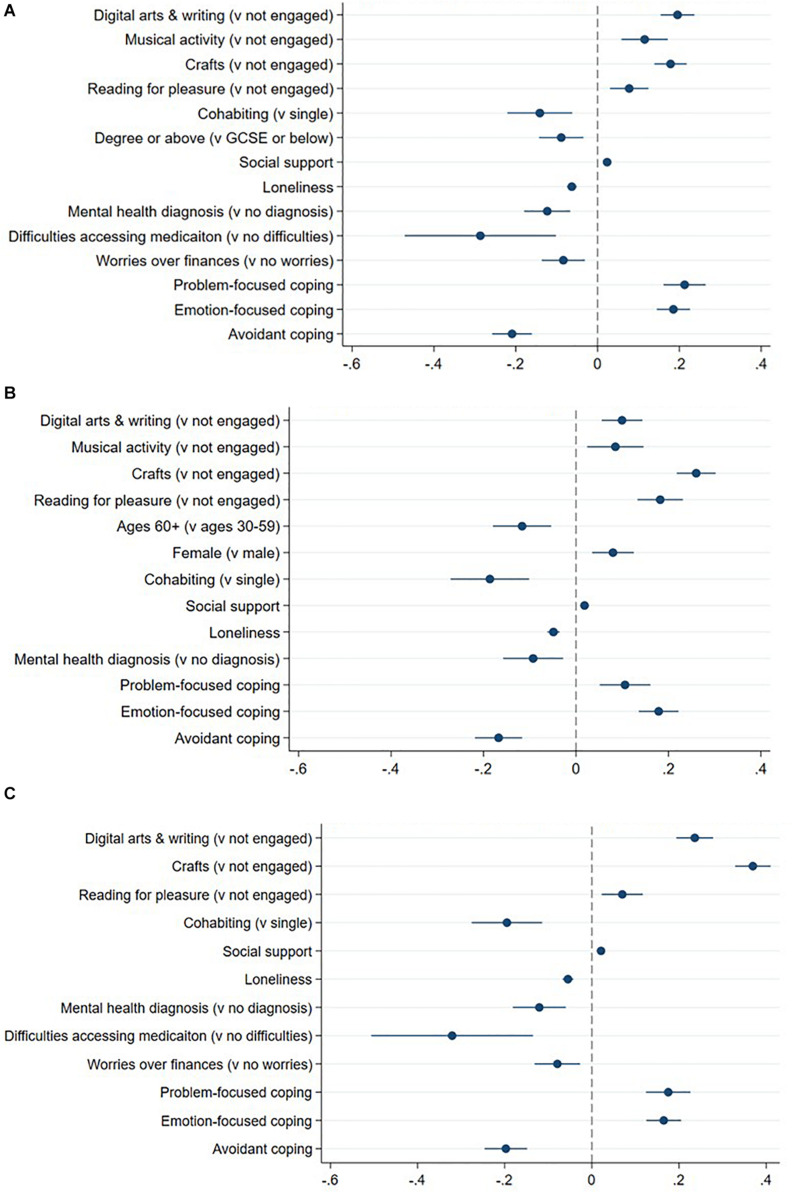
**(A)** Approach strategy through arts activities. Panel **(A)** shows coefficients and 95% CIs from OLS regression; the model was adjusted for all covariates. Only results with *p* < 0.01 are shown. **(B)** Avoidance strategy through arts activities. Panel **(B)** shows coefficients and 95% CIs from OLS regression; the model was adjusted for all covariates. Only results with *p* < 0.01 are shown. **(C)** Self-development strategy through arts activities. Panel **(C)** shows coefficients and 95% CIs from OLS regression; the model was adjusted for all covariates. Only results with *p* < 0.01 are shown.

Finally, we explored how different arts activities were used to regulate emotions during lockdown. Results show that respondents who engaged in any of the four arts activities also reported of using them as approach and avoidance strategies to help cope with their emotions. Respondents who engaged in digital arts and writing, crafts, and reading for pleasure were also more likely to use these activities to improve their self-development (Figures 3A–C).

#### Demographic Factors

Older people (aged 60+) were less likely to use arts to avoid negative emotions, but age was otherwise unrelated to use of ERS when engaging in the arts. Females were more likely to use arts to help them avoid negative emotions, whereas people who were single and never married were more likely than those living with a partner to use the arts to regulate their emotions. Ethnicity, living arrangement and living areas were not associated with the use of ERS when engaging in the arts (Figures 3A–C).

#### Socio-Economic Position

People with a degree or above qualification were less likely to use the arts to approach their problems. Employment, living space, keyworker status, household income and home ownership were not related to the use of ERS when engaging in the arts (Figures 3A–C).

#### Psychosocial and Health Factors

While individuals with higher levels of social support were likely to use arts for all ERS, people who were lonelier or had a mental health diagnosis were less likely to use ERS when engaging in the arts. Social network and physical health diagnosis were not associated with the use of ERS when engaging in the arts (Figures 3A–C).

#### Adverse Events/Worries

People who were unable to access required medication or those who were worried about finances during lockdown were less likely to use the arts to approach problems or for self-development strategy. No associations were found for other adverse events or worries (Figures 3A–C).

#### Coping Styles

Finally, individuals with a problem-focused or emotion-focused coping style were more likely to use arts for all ERS. However, people with an avoidant coping style were less likely to use ERS when engaging in the arts. No associations were found between the supportive coping style and the use of ERS (Figures 3A–C).

## Discussion

Our analysis suggests that there were four main types of home-based arts engagement during the COVID-19 pandemic: digital arts and writing, musical activities, crafts, and reading for pleasure. The strongest predictors of home-based arts engagement were age, educational attainment, social support, and emotion-focused or supportive coping styles, which were associated with all types of art forms. Further, age, gender and educational attainment appear to have the strongest effects. Overall, 62% of respondents continued with the same amount of home arts engagement as prior to COVID-19, but 22% of respondents reported increasing their arts engagement during lockdown, and 52% of these either maintained high levels of engagement or further increased their arts engagement 3 months later after lockdown had eased. Conversely, 16% of respondents reported that their arts engagement decreased during lockdown, and of these, 51% reported either remained low levels of engagement or had further decreased in arts engagement 3 months later. All arts activities were used as approach and avoidance strategies to help people cope with their emotions, while digital arts and writing, crafts, and reading for pleasure were additionally used to help improve respondents’ self-development.

### Predictors of Changing Patterns of Arts Engagement

When comparing the arts engagement during the pandemic to pre-pandemic circumstance, we found that some factors identified as predictors of arts engagement in RQ1 and RQ2 align with previous literature on predictors of arts engagement prior to COVID-19 (Devine and Dowds, 2013; Parkinson et al., 2014; Mak et al., 2020b). For instance, young people, females, people living in rural areas, those with higher educational levels, and people with higher levels of perceived social support and with greater social network are more likely to engage in the arts (Devine and Dowds, 2013; Parkinson et al., 2014; Risner, 2014; Mak et al., 2020a, b). In particular, younger adults and respondents with greater social support were likely to have increased their engagement during the pandemic. Further, our study also shows that adults living with children were less likely to read for pleasure during the pandemic [as has also been shown in a previous study (Mak et al., 2020b)]. However, we also found a lot of variation in arts engagement during the pandemic, in relation to respondents’ ethnicity, partnership status, SES, levels of loneliness, and mental and physical health conditions, as well as new emerging factors in the engagement during lockdown, including adverse events and worries, and people’s own coping styles.

The patterns and predictors of arts engagement before and during the pandemic can be understood through the lens of the COM-B behavior change model, which suggests that engagement in activities is influenced by people’s *capabilities* (i.e., knowledge and skills), *opportunities* (i.e., individuals’ social and environmental), and *motivations* (i.e., habitual processes, emotional responding and analytical decision-making) (COM-B) (Michie et al., 2011). For example, younger adults may have greater levels of *psychological capabilities* and *environmental opportunities* to access to internet and engage in a variety of arts activities at home (e.g., virtual choir), whereas older adults are perhaps more likely to engage in the arts in a group and the lockdown measures might have prevented them from doing so. It is also possible that younger adults who experienced poorer mental health across lockdown and were more likely to have been furloughed or lost their job (Fancourt et al., 2020) might have been more *motivated* to make conscious efforts to engage in leisure activities. On the other hand, adults living with children may have had fewer *physical opportunities* to do arts activities (most likely due to time constraints) regardless of the pandemic circumstances.

While some of the demographic factors remain unaffected during the pandemic, our study shows that ethnicity played a role in the changing patterns of arts engagement. In contrast to previous research, which shows that people of ethnic minority background were in general less likely to engage in the arts (Parkinson et al., 2014; Mak et al., 2020b), we found no ethnic differences during the pandemic. One possible explanation is that people from white ethnic backgrounds may have been more likely to engage in arts activities within community venues prior to the pandemic. Therefore, with the sudden change in *physical* and *environmental opportunities* with only home-based engagement available during lockdown, the ethnic difference in participation rate may have been reduced. Limited *opportunities* may also be reflected in people with different partnership status during the pandemic. Whilst previous studies show that non-married individuals were usually more likely to participate in the arts than those who were married (Mak et al., 2020b), we found that single and never married read less than cohabiting couples but engaged more in digital arts and writing activities than those who were divorced or widowed (possibly due to increased *opportunities* in online activities).

Individual socioeconomic position was also related to changing patterns of the engagement during the pandemic. Previous studies have suggested that people from lower SES generally engaged less in the arts prior to the pandemic (Parkinson et al., 2014; Mak et al., 2020b), yet we found no socio-economic differences during the lockdown. One explanation is that the sudden transition to online and remote arts forms may have created new *environmental and physical opportunities* for people from various background to engage in the arts. Further, the strict lockdown rules might have reduced *physical opportunities* for people with higher SES to engage in the arts due to cancelation of arts events, programs and classes, and hence the socio-economic differences in engagement rate may have been reduced. However, it is also plausible that the gradient in participation across levels of SES were found more prominently in outdoor arts activities (e.g., attending performances, paid group memberships, and courses) where people with advantaged backgrounds are most likely to be able to afford participation. Home-based arts activities, on the other hand, are perhaps more commonly engaged by people across various SES due to easy accessibility to arts materials (e.g., coloring pencils, books, and sketchbooks). Moreover, a rapid increase in activities such as rainbow drawing to support frontline health professionals and keyworkers in the United Kingdom may also have provided *motivations* for people with different backgrounds to engage in the arts. Additionally, when comparing the amount of arts engagement across levels of SES, our study shows that individuals who were economically inactive were less likely to have increased their engagement than those who were unemployed and seeking work. This may suggest that people who were looking for jobs during the pandemic may have had increased *motivations* for arts engagement for emotional regulation and relaxation purposes. Our analysis also found that household income, keyworker status and home ownership were factors predicting people’s arts engagement during lockdown, most likely due to new and emerging *physical opportunities* (e.g., time constraints). However, it appears that the pandemic may not have affected the engagement rate amongst people with higher education level. A possible explanation for this is that people with higher education levels are likely to have greater psychological *capabilities* to engage in the arts (e.g., musical skills and confidence) (Fancourt and Baxter, 2020), and hence were able to maintain high levels of engagement even during a national lockdown.

The unchanged pattern of education and arts engagement is also reflected in people’s social support and network, where greater levels of support and network were associated with higher arts participation rate both prior to and during the pandemic (Risner, 2014). Social support and network may have affected people’s *psychological capabilities* (e.g., skills exchange) and *motivations* (e.g., social belongings) to involve in arts activities through group engagement. However, the current pandemic may have also created new *environmental opportunities* and *motivations* for people with higher levels of loneliness and a diagnosed mental condition to engage in the arts, particularly in digital arts and writing and in crafts activities. This is the opposite finding to some previous research, which has suggested that happier people are more likely to engage in the arts (Fancourt and Baxter, 2020), and could suggest that the proliferation and encouragement of online and home-based activities helped to reduce barriers to access the arts. Similarly, our study shows that there were no differences in arts engagement between people with or without a physical health condition. In contrast to the previous findings (Parkinson et al., 2014), greater accessibility to and availability of online arts engagement may have helped wider audiences to engage in arts activities, creating more *environmental and physical* opportunities for people (including those with a physical health condition) who have traditionally engaged less in the arts.

Finally, we also looked at specific experiences during the pandemic to explore whether they may play a role in people’s arts engagement. We found that individuals who had lost work, or were worried about catching COVID-19 or their personal safety engage more digital arts and writing and musical activities. It is possible that adverse events and worries might have triggered higher *motivations* to engage with the arts as part of emotion-focused and supportive coping styles to regulate their emotional responses to these events/worries. Indeed, in our final analysis, we found that those with an emotion-focused coping style were more likely to engage in any kinds of activities and make greater use of the arts to regulate their emotions. We also found that both problem-focused and supportive coping styles were positively associated with most of the arts activities, and in particular, people with a problem-focused coping style tended to use the arts to manage their emotions. This echoes previous literature that shows that a problem-focused coping style usually involve directly addressing and attempting to mitigate stressors (Aspinwall and Taylor, 1997). However, as there is little control an individual can have over the wider pandemic, these findings indicate that these individuals may have focused on improving their own mental health during lockdown through engaging in activities such as the arts. Further, emotion-focused coping styles seek to minimize emotional responses to stressors (Baker and Berenbaum, 2007), therefore individuals who favor these coping styles may seek out activities, such as the arts, which are known to have mental health benefits. In contrast, those with an avoidant coping style engaged less in arts activities during lockdown than before. Avoidant coping styles are used when individuals wish to avoid and ignore the stressor rather than taking action (Rippetoe and Rogers, 1987; Skinner et al., 2003), so could indicate that individuals were avoiding acknowledging the impact lockdown had on their own mental and physical health and therefore were less likely to seek out activities such as the arts as a means to improve wellbeing.

### Arts and Regulation of Emotions

In our final analysis, we examined how different arts activities were used to regulate emotions during lockdown (RQ3). Our results show that all arts activities explored were used as approach and avoidance strategies to help cope with their emotions, while digital arts and writing, crafts, and reading for pleasure additionally helped improve people’s self-development. We also found some variations in regulating emotions through the arts by personal characteristics. For example, while females were more likely to use arts to avoid negative emotions, people who were single and never married were more likely than those living with a partner to use the arts to regulate their emotions. People who were lonelier or had a mental health diagnosis were less likely to use ERS when engaging in the arts. Overall, it is promising that arts appeared to help people with regulation of their emotions during this time, and these findings may help explain previous work during COVID-19 lockdown that suggests arts engagement was associated with better mental health (Bu et al., 2020).

Our study is one of the first studies that examined the predictors and patterns of arts engagement during the COVID-19 pandemic, as well as the implications of the engagement on emotional regulations. The analysis was based on a large, heterogeneous sample across all major socio-demographic groups and the analyses were weighed to population proportions. However, this study was not without limitations. First, whilst our data was weighted to proportion of age, gender, ethnicity, education and country of living) obtained from the Office for National Statistics (2020), it is possible that there might be other characteristics related to survey response were not being accounted for in the weighting procedure. Second, we only explored home-based arts activities during lockdown and were conscious that people’s engagement in community arts and broader cultural activities was curtailed by the onset of the pandemic. So the net amount of arts engagement for individuals may well have been reduced overall. Nevertheless, our findings give an insight into changing patterns of home-based activities. Relatedly, the groupings for arts activities were not an indication of definitive categories. Instead they were grouped based on the correlations amongst these activities. Therefore, it is possible that using an alternate statistical technique or alternative measures of arts engagement could have led to a different set of groupings. However, the types of arts activities suggested are in line with those suggested in previous studies (e.g., Mak et al., 2020b). Notably, to capture various forms of arts engagement during lockdown, we did not specify whether respondents were engaged in person or virtually. Future work would be needed to explore the patterns and predictors of online arts engagement, as well as the impact on emotional regulation. Third, as our analysis was based on cross-sectional data, causality cannot be established. It is plausible that for some predictors like loneliness the relationship was more bi-directional rather than uni-directional, with arts engagement possibly being a gateway to social interactions through online or digital communities and helping to reduce loneliness. Given the socio-demographic factors were asked in the first wave of the study (i.e., prior to the lockdown), we did not examine how participation in government schemes such as furlough schemes might have affected people’s time on leisure activities at home. Moreover, given that gender is a strong predictor estimating arts engagement levels, it would be interesting to also examine whether the levels would vary across gender categories that are non-binary. Finally, future research is needed to investigate the way people structured their time (e.g., work, housework and childcare) during lockdown and how this may have affected their arts engagement. More research is also required to examine whether the positive benefits of arts activities in everyday life shown in previous studies continued to benefit people during the lockdown measure where social lives are curtailed.

## Conclusion

Overall, this study suggests that while individuals with certain characteristics had similar levels of arts engagement before and during the COVID-19 pandemic, there was also some heterogeneity across social, cultural and economic groups. Our findings could be understood through the lens of COM-B behavioral change model (capabilities, opportunities, and motivations), in which the pandemic may have created new opportunities and motivations for people who have been traditionally excluded from the arts to engage while supporting others (possibly those with higher initial capabilities to engage) to maintain their usual levels of engagement. We further identified some factors that emerged as more prominent predictors of engagement during the pandemic, including worries about adverse events experienced due to COVID-19 and individual coping styles. These predictors may have increased people’s motivations to engage in the arts. Additionally, this study shows how arts activities were used during lockdown to help individuals manage their emotions. While more studies are needed to understand the motivations and barriers of arts engagement during emergency and normal circumstances, this study suggests that there may have been different dynamics in social, cultural and economic patterning of arts engagement, leading to some new patterns in how people engaged in artistic activities and the impact this had on them during the pandemic situation. Future research is encouraged to explore how these changing audience profiles for the arts develop as the pandemic continues and in its aftermath to ascertain whether new audiences to the arts during COVID-19 continue as audiences in the future.

## Data Availability Statement

Anonymous data will be made available following the end of the pandemic.

## Ethics Statement

Ethical approval for the COVID-19 Social Study was granted by the UCL Ethics Committee. All participants provided fully informed consent. The study is GDPR compliant.

## Author Contributions

HWM conducted the data management and data analyses and provided input on the manuscript. MF and DF assisted with analytical issues and provided input on the analytical scheme and the manuscript. DF designed the study. All authors are responsible for reported research, analysis and interpretation of data, and drafted and revised of the manuscript.

## Conflict of Interest

The authors declare that the research was conducted in the absence of any commercial or financial relationships that could be construed as a potential conflict of interest.

## Footnotes

1 https://github.com/UCL-BSH/CSSUserGuide
