# Supplementary material for: Predictors and Impact of Arts Engagement During the COVID-19 Pandemic: Analyses of Data From 19,384 Adults in the COVID-19 Social Study
Source: Front Psychol. 2021 Apr 26;12:626263. doi: 10.3389/fpsyg.2021.626263 (PMC8109242; doi:10.3389/fpsyg.2021.626263)
Supplement: Supplementary file 1 [file Table_1.DOCX]

# Supplementary Materials

| **Supplementary Table 1: Frequency of home-based arts engagement with the original 5-point scale (raw data; with weight)** | | | | | |
| --- | --- | --- | --- | --- | --- |
|  | Not at all | A few days | Once or twice a week | Most days | Every day |
| Singing (N=32,593) | 79.2% | 10.3% | 4.74% | 4.18% | 1.61% |
| Playing a musical instrument (N=32,436) | 89.7% | 4.69% | 2.26% | 2.28% | 1.03% |
| Painting, drawing, printmaking or sculpture (N=32,345) | 87.1% | 7.83% | 3.42% | 1.30% | 0.37% |
| Reading books, stories or poetry (N=32,656) | 32.9% | 20.9% | 10.4% | 19.0% | 16.8% |
| Textile crafts e.g. embroidery, crocheting or knitting (N=32,522) | 84.9% | 6.10% | 3.37% | 3.45% | 2.22% |
| Wood crafts e.g. carving or furniture making (N=32,435) | 95.8% | 2.68% | 1.11% | 0.36% | 0.00% |
| Other crafts e.g. pottery, calligraphy or jewellery making (N=32,521) | 95.8% | 2.43% | 1.11% | 0.48% | 0.16% |
| Creative writing (N=32,611) | 92.1% | 4.56% | 1.64% | 1.05% | 0.63% |
| Dancing (N=32,520) | 88.6% | 6.73% | 3.26% | 1.05% | 0.37% |
| Photography (N=32,392) | 78.4% | 12.8% | 5.48% | 2.43% | 0.83% |
| Creating digital artworks or animations (N=32,370) | 96.8% | 2.07% | 0.70% | 0.37% | 0.00% |
| Making films or videos (N=32,376) | 94.2% | 3.93% | 1.31% | 0.42% | 0.12% |
| Listening to music (N=32,603) | 18.5% | 20.3% | 13.9% | 23.9% | 23.5% |
| Other creative activity (N=31,661) | 75.8% | 10.3% | 6.54% | 4.32% | 3.05% |

| **Supplementary Table 2: Validation of the Emotion Regulation Strategies for Artistic Creative Activities Scale (ERS-ACA). Items included in the ERS-ACA scale with factor loadings.** | | | |
| --- | --- | --- | --- |
|  | **Approach strategy (alpha=0.9024)** | **Avoidance strategy (alpha=0.9004)** | **Self-development strategy (alpha=0.9017)** |
| When you are engaging in these activities, how much do you agree with the statement below: |  |  |  |
| I can contemplate what is going on in my life with a clear mind | 0.3744 |  |  |
| It helps me refocus on what matter in my life | 0.5774 |  |  |
| It helps me to come to terms with my own emotions | 0.7129 |  |  |
| It helps to put worries or problems I have in perspective | 0.7094 |  |  |
| It helps to understand my own feelings on things that are on my mind | 0.7991 |  |  |
| It makes me reflect on my emotions | 0.7414 |  |  |
| I can block out any unwanted thoughts or feelings |  | 0.6452 |  |
| I can shake off any anxieties in my life |  | 0.5909 |  |
| I feel I am in my own little bubble, away from ordinary worries |  | 0.5827 |  |
| It helps me forget about my worries |  | 0.6856 |  |
| It helps to disengage from things that are bothering me |  | 0.7188 |  |
| It makes me feel detached from negative things in my life |  | 0.6090 |  |
| It redirects my attention so I forget unwanted thoughts and feelings |  | 0.6865 |  |
| I feel more confident in myself |  |  | 0.7309 |
| It boosts my self-esteem |  |  | 0.7648 |
| It gives me a sense of purpose |  |  | 0.6177 |
| It makes me feel stronger in myself |  |  | 0.5203 |
| It reaffirms my identity |  |  | 0.4259 |
| Note: All items scored from 1(strongly disagree) to 5 (strongly agree). Factors loadings were produced by orthogonal rotation. The approach and avoidance strategies have a correlation coefficient of 0.66; the approach and self-development strategies have a correlation coefficient of 0.77; and the avoidance and self-development strategies have a correlation coefficient of 0.69. Factor loadings indicated here are in line with those shown in the previous ERS-ACA validation study, which consists of the complete measure and instructions: Fancourt D, Garnett C, Spiro N, West R, Müllensiefen D. How do artistic creative activities regulate our emotions? Validation of the Emotion Regulation Strategies for Artistic Creative Activities Scale (ERS-ACA). PLoS One. 2019;14(2):e0211362. Available from: <https://dx.plos.org/10.1371/journal.pone.0211362>. | | | |

| Supplementary Table 3: Comparison of items in the original and revised Perceived Social Support Questionnaire (F-SozU K-6) | |
| --- | --- |
| Original | Adapted for COVID-19  In the past week, I feel… |
| I experience a lot of understanding and security from others | I have experienced a lot of understanding and support from others |
| I know a very close person whose help I can always count on | I have a very close person whose help I can always count on |
| If necessary, I can easily borrow something I might need from neighbours or friends | If necessary, I can easily borrow something I need from neighbours or friends |
| I know several people with whom I like to do things | I have people with whom I can spend time and do things together |
| When I am sick, I can without hesitation ask friends and family to take care of  important matters for me | If I get sick, I have friends and family who will take care of me |
| If I am down, I know to whom I can go without hesitation | If I am feeling down, I have people I can talk to without hesitation |

| **Supplementary Table 4: Average marginal effects of predictors for the types of arts activities during the COVID-19 pandemic in the UK (weighted; N=19,384)** | | | | | | | | | | | | |
| --- | --- | --- | --- | --- | --- | --- | --- | --- | --- | --- | --- | --- |
|  | **Digital arts & writing** | | | **Musical activities** | | | **Crafts** | | | **Reading for pleasure** | | |
|  | **AME** | **95% CI** | **P-value** | **AME** | **95% CI** | **P-value** | **AME** | **95% CI** | **P-value** | **AME** | **95% CI** | **P-value** |
| *Model 1: Demographic backgrounds* | | | |  | |  |  |  |  |  |  |  |
| Ages 18-29 | 0.09 | 0.05 - 0.14 | 0.000 | 0.06 | 0.03 - 0.08 | 0.000 | 0.09 | 0.05 - 0.14 | 0.000 | 0.06 | 0.01 - 0.10 | 0.015 |
| Ages 60+ | -0.01 | -0.03 - 0.01 | 0.251 | -0.08 | -0.10 - -0.06 | 0.000 | 0.03 | 0.01 - 0.06 | 0.007 | 0.11 | 0.09 - 0.14 | 0.000 |
| (ref: ages 30-59) |  |  |  |  |  |  |  |  |  |  |  |  |
| Female | 0.00 | -0.02 - 0.02 | 0.740 | -0.02 | -0.03 - -0.00 | 0.021 | 0.19 | 0.17 - 0.21 | 0.000 | 0.17 | 0.15 - 0.19 | 0.000 |
| (ref: male) |  |  |  |  |  |  |  |  |  |  |  |  |
| White ethnic | -0.01 | -0.06 - 0.04 | 0.656 | -0.04 | -0.08 - 0.01 | 0.105 | 0.06 | 0.01 - 0.10 | 0.028 | 0.06 | 0.02 - 0.11 | 0.010 |
| (ref: ethnic minority) |  |  |  |  |  |  |  |  |  |  |  |  |
| Divorced or widowed | -0.06 | -0.09 - -0.02 | 0.001 | 0.00 | -0.03 - 0.04 | 0.773 | -0.04 | -0.08 - -0.01 | 0.021 | -0.03 | -0.08 - 0.01 | 0.103 |
| In a relationship/married but living apart | -0.02 | -0.07 - 0.03 | 0.391 | 0.04 | 0.00 - 0.08 | 0.030 | 0.01 | -0.04 - 0.06 | 0.722 | 0.00 | -0.05 - 0.06 | 0.953 |
| In a relationship/married and cohabiting | -0.04 | -0.07 - -0.00 | 0.046 | 0.01 | -0.02 - 0.05 | 0.378 | 0.01 | -0.03 - 0.05 | 0.600 | 0.05 | 0.01 - 0.09 | 0.009 |
| (ref: Single and never married) |  |  |  |  |  |  |  |  |  |  |  |  |
| Not living alone & without children | 0.01 | -0.02 - 0.05 | 0.521 | -0.01 | -0.04 - 0.01 | 0.315 | 0.03 | -0.00 - 0.07 | 0.088 | -0.02 | -0.05 - 0.02 | 0.384 |
| Not living alone & with children | -0.01 | -0.05 - 0.03 | 0.688 | -0.03 | -0.06 - 0.01 | 0.148 | 0.01 | -0.03 - 0.05 | 0.549 | -0.06 | -0.11 - -0.02 | 0.003 |
| (ref: living alone) |  |  |  |  |  |  |  |  |  |  |  |  |
| Living in village/hamlet/isolated dwelling | 0.05 | 0.03 - 0.07 | 0.000 | -0.01 | -0.03 - 0.00 | 0.132 | 0.02 | -0.01 - 0.04 | 0.145 | -0.00 | -0.03 - 0.02 | 0.875 |
| (ref: living in city/town) |  |  |  |  |  |  |  |  |  |  |  |  |
| *Model 2: Model 1 + Socio-economic position* | | | |  |  |  |  |  |  |  |  |  |
| Full-time employment/self-employed | -0.04 | -0.12 - 0.03 | 0.237 | 0.03 | -0.04 - 0.09 | 0.409 | -0.04 | -0.11 - 0.04 | 0.346 | -0.03 | -0.10 - 0.04 | 0.432 |
| Part-time employment | -0.04 | -0.12 - 0.03 | 0.259 | 0.01 | -0.05 - 0.08 | 0.707 | -0.02 | -0.10 - 0.06 | 0.636 | 0.02 | -0.05 - 0.09 | 0.598 |
| Economically inactive (incl. student/ retired/ homemakers/ unable to work due to disability) | -0.05 | -0.13 - 0.02 | 0.152 | -0.01 | -0.07 - 0.06 | 0.768 | -0.02 | -0.10 - 0.05 | 0.530 | -0.00 | -0.07 - 0.07 | 0.931 |
| (ref: unemployed & seeking work) |  |  |  |  |  |  |  |  |  |  |  |  |
| Post-16 vocational or A-levels qualifications or equivalent | 0.11 | 0.08 - 0.13 | 0.000 | 0.03 | 0.01 - 0.06 | 0.003 | 0.04 | 0.01 - 0.07 | 0.009 | 0.10 | 0.07 - 0.13 | 0.000 |
| Degree or above | 0.17 | 0.15 - 0.20 | 0.000 | 0.05 | 0.03 - 0.07 | 0.000 | 0.11 | 0.09 - 0.14 | 0.000 | 0.22 | 0.20 - 0.25 | 0.000 |
| (ref: GCSE/CSE/O-levels or equivalent or below) |  |  |  |  |  |  |  |  |  |  |  |  |
| Household income >£30,000 | 0.01 | -0.02 - 0.03 | 0.501 | 0.01 | -0.01 - 0.03 | 0.513 | -0.04 | -0.06 - -0.01 | 0.004 | 0.02 | -0.00 - 0.05 | 0.064 |
| (ref: household income <£30,000) |  |  |  |  |  |  |  |  |  |  |  |  |
| Not living in overcrowded households | -0.01 | -0.05 - 0.02 | 0.509 | 0.02 | -0.01 - 0.05 | 0.140 | 0.00 | -0.04 - 0.04 | 0.885 | 0.02 | -0.02 - 0.06 | 0.309 |
| (ref: living in overcrowded households) |  |  |  |  |  |  |  |  |  |  |  |  |
| Non-keyworkers | 0.07 | 0.05 - 0.09 | 0.000 | 0.00 | -0.02 - 0.02 | 0.885 | 0.06 | 0.03 - 0.08 | 0.000 | 0.06 | 0.03 - 0.08 | 0.000 |
| (ref: keyworkers) |  |  |  |  |  |  |  |  |  |  |  |  |
| House owners | -0.04 | -0.07 - -0.02 | 0.001 | -0.01 | -0.03 - 0.01 | 0.571 | -0.02 | -0.04 - 0.01 | 0.220 | 0.04 | 0.01 - 0.06 | 0.003 |
| (ref: not house owners) |  |  |  |  |  |  |  |  |  |  |  |  |
| *Model 3: Model 2 + psychosocial wellbeing & health conditions* | | | |  |  |  |  |  |  |  |  |  |
| Social support | 0.00 | 0.00 - 0.01 | 0.000 | 0.01 | 0.00 - 0.01 | 0.000 | 0.01 | 0.00 - 0.01 | 0.000 | 0.00 | 0.00 - 0.01 | 0.000 |
| Large social network (≥3 friends) | 0.03 | 0.00 - 0.05 | 0.025 | 0.04 | 0.02 - 0.05 | 0.000 | 0.03 | 0.00 - 0.05 | 0.021 | 0.05 | 0.03 - 0.07 | 0.000 |
| Loneliness | 0.01 | 0.00 - 0.01 | 0.005 | 0.00 | -0.00 - 0.01 | 0.112 | 0.00 | -0.00 - 0.01 | 0.460 | -0.01 | -0.01 - -0.00 | 0.015 |
| Diagnosed mental health condition | 0.01 | -0.02 - 0.04 | 0.428 | 0.00 | -0.02 - 0.03 | 0.702 | 0.05 | 0.02 - 0.08 | 0.000 | -0.01 | -0.04 - 0.02 | 0.567 |
| Diagnosed physical health condition or disability | -0.00 | -0.03 - 0.02 | 0.627 | -0.02 | -0.04 - -0.00 | 0.013 | -0.01 | -0.03 - 0.01 | 0.411 | -0.01 | -0.03 - 0.01 | 0.526 |
| *Model 4: Model 3 + adverse events/worries* |  |  |  |  |  |  |  |  |  |  |  |  |
| Adverse events |  |  |  |  |  |  |  |  |  |  |  |  |
| COVID-19 diagnosis | 0.03 | 0.00 - 0.06 | 0.031 | 0.01 | -0.02 - 0.03 | 0.681 | 0.02 | -0.01 - 0.05 | 0.239 | 0.03 | -0.00 - 0.06 | 0.075 |
| Physically/psychologically abused | 0.04 | -0.01 - 0.09 | 0.090 | -0.02 | -0.06 - 0.01 | 0.218 | -0.02 | -0.07 - 0.03 | 0.493 | -0.02 | -0.07 - 0.03 | 0.407 |
| Financial difficulties | 0.01 | -0.03 - 0.04 | 0.769 | -0.01 | -0.04 - 0.02 | 0.614 | 0.03 | -0.01 - 0.07 | 0.095 | -0.02 | -0.05 - 0.02 | 0.267 |
| Lost work | 0.09 | 0.04 - 0.13 | 0.000 | 0.03 | -0.02 - 0.07 | 0.251 | 0.05 | -0.01 - 0.10 | 0.097 | 0.02 | -0.03 - 0.07 | 0.485 |
| Difficulties accessing food | 0.00 | -0.10 - 0.10 | 0.989 | 0.01 | -0.06 - 0.08 | 0.792 | -0.06 | -0.15 - 0.04 | 0.249 | 0.02 | -0.07 - 0.11 | 0.710 |
| Difficulties accessing medication | -0.04 | -0.13 - 0.05 | 0.394 | 0.02 | -0.05 - 0.09 | 0.607 | 0.03 | -0.07 - 0.13 | 0.551 | -0.03 | -0.11 - 0.06 | 0.554 |
| Worries |  |  |  |  |  |  |  |  |  |  |  |  |
| Catching COVID-19 | 0.03 | 0.01 - 0.05 | 0.001 | 0.02 | 0.01 - 0.04 | 0.006 | 0.02 | 0.00 - 0.04 | 0.034 | -0.01 | -0.04 - 0.01 | 0.187 |
| Personal safety | 0.04 | 0.01 - 0.07 | 0.008 | -0.00 | -0.03 - 0.03 | 0.889 | -0.00 | -0.04 - 0.03 | 0.936 | 0.00 | -0.03 - 0.04 | 0.809 |
| Finances | 0.01 | -0.02 - 0.03 | 0.502 | 0.01 | -0.01 - 0.03 | 0.410 | 0.03 | 0.01 - 0.06 | 0.019 | -0.01 | -0.04 - 0.01 | 0.342 |
| Unemployment | 0.02 | -0.01 - 0.05 | 0.165 | -0.00 | -0.03 - 0.03 | 0.939 | -0.00 | -0.04 - 0.03 | 0.874 | 0.02 | -0.02 - 0.05 | 0.311 |
| Food access | 0.00 | -0.04 - 0.04 | 0.987 | -0.02 | -0.06 - 0.01 | 0.111 | 0.02 | -0.03 - 0.06 | 0.429 | -0.02 | -0.06 - 0.02 | 0.343 |
| Medication access | 0.05 | 0.00 - 0.10 | 0.044 | -0.01 | -0.05 - 0.03 | 0.596 | 0.03 | -0.02 - 0.08 | 0.292 | 0.01 | -0.04 - 0.06 | 0.715 |
| *Model 5: Model 4 + coping styles* |  |  |  |  |  |  |  |  |  |  |  |  |
| Problem-focused coping | 0.05 | 0.03 - 0.08 | 0.000 | 0.02 | 0.00 - 0.05 | 0.025 | 0.09 | 0.06 - 0.11 | 0.000 | 0.02 | -0.00 - 0.05 | 0.081 |
| Emotion-focused coping | 0.05 | 0.03 - 0.06 | 0.000 | 0.03 | 0.01 - 0.05 | 0.000 | 0.04 | 0.02 - 0.05 | 0.000 | 0.04 | 0.02 - 0.06 | 0.000 |
| Avoidant coping | -0.02 | -0.04 - 0.00 | 0.055 | -0.01 | -0.03 - 0.01 | 0.257 | -0.01 | -0.04 - 0.01 | 0.190 | -0.02 | -0.04 - 0.00 | 0.108 |
| Supportive coping | 0.05 | 0.03 - 0.06 | 0.000 | 0.04 | 0.02 - 0.05 | 0.000 | 0.03 | 0.01 - 0.05 | 0.007 | 0.05 | 0.03 - 0.07 | 0.000 |

| **Supplementary Table 5: Sensitivity analysis predicting musical activities (without “listening to music” activity) (weighted; N=19,317)** | | | |
| --- | --- | --- | --- |
|  | **Musical activities** | | |
|  | **OR** | **95% CI** | **P-value** |
| Ages 18-29 | 2.28 | 1.88 - 2.76 | 0.000 |
| Ages 60+ | 0.75 | 0.68 - 0.84 | 0.000 |
| (ref: ages 30-59) |  |  |  |
| Female | 1.74 | 1.57 - 1.93 | 0.000 |
| (ref: male) |  |  |  |
| White ethnic | 0.73 | 0.58 - 0.91 | 0.004 |
| (ref: ethnic minority) |  |  |  |
| Divorced or widowed | 0.75 | 0.63 - 0.90 | 0.001 |
| In a relationship/married but living apart | 0.99 | 0.79 - 1.24 | 0.915 |
| In a relationship/married and cohabiting | 0.80 | 0.68 - 0.94 | 0.008 |
| (ref: Single and never married) |  |  |  |
| Not living alone & without children | 1.13 | 0.96 - 1.33 | 0.150 |
| Not living alone & with children | 1.44 | 1.20 - 1.73 | 0.000 |
| (ref: living alone) |  |  |  |
| Living in village/hamlet/isolated dwelling | 1.01 | 0.90 - 1.12 | 0.917 |
| (ref: living in city/town) |  |  |  |
| Constant | 0.44 | 0.34 - 0.57 | 0.000 |
| *Model 2: Model 1 + Socio-economic position* | | |  |
| Full-time employment/self-employed | 1.02 | 0.72 - 1.46 | 0.895 |
| Part-time employment | 1.08 | 0.75 - 1.57 | 0.678 |
| Economically inactive (incl. student/ retired/ homemakers/ unable to work due to disability) | 1.03 | 0.72 - 1.49 | 0.865 |
| (ref: unemployed & seeking work) |  |  |  |
| Post-16 vocational or A-levels qualifications or equivalent | 1.30 | 1.12 - 1.51 | 0.001 |
| Degree or above | 1.78 | 1.56 - 2.03 | 0.000 |
| (ref: GCSE/CSE/O-levels or equivalent or below) |  |  |  |
| Household income >£30,000 | 1.03 | 0.91 - 1.15 | 0.681 |
| (ref: household income <£30,000) |  |  |  |
| Not living in overcrowded households | 0.88 | 0.73 - 1.05 | 0.165 |
| (ref: living in overcrowded households) |  |  |  |
| Non-keyworkers | 1.10 | 0.97 - 1.24 | 0.150 |
| (ref: keyworkers) |  |  |  |
| House owners | 0.94 | 0.83 - 1.06 | 0.308 |
| (ref: not house owners) |  |  |  |
| Constant | 0.30 | 0.19 - 0.48 | 0.000 |
| *Model 3: Model 2 + psychosocial wellbeing & health conditions* | | |  |
| Social support | 1.04 | 1.03 - 1.05 | 0.000 |
| Large social network (≥3 friends) | 1.15 | 1.03 - 1.30 | 0.018 |
| Loneliness | 1.03 | 1.00 - 1.06 | 0.025 |
| Diagnosed mental health condition | 1.00 | 0.88 - 1.14 | 0.990 |
| Diagnosed physical health condition or disability | 0.99 | 0.89 - 1.09 | 0.804 |
| Constant | 0.14 | 0.08 - 0.25 | 0.000 |
| *Model 4: Model 3 + adverse events/worries* |  |  |  |
| Adverse events |  |  |  |
| COVID-19 diagnosis | 1.28 | 1.10 - 1.48 | 0.001 |
| Physically/psychologically abused | 1.14 | 0.90 - 1.44 | 0.265 |
| Financial difficulties | 0.94 | 0.79 - 1.11 | 0.447 |
| Lost work | 1.18 | 0.92 - 1.50 | 0.195 |
| Difficulties accessing food | 0.76 | 0.47 - 1.22 | 0.256 |
| Difficulties accessing medication | 1.06 | 0.67 - 1.66 | 0.814 |
| Worries |  |  |  |
| Catching COVID-19 | 1.05 | 0.95 - 1.16 | 0.356 |
| Personal safety | 1.10 | 0.93 - 1.31 | 0.274 |
| Finances | 1.06 | 0.94 - 1.21 | 0.343 |
| Unemployment | 1.00 | 0.85 - 1.18 | 0.989 |
| Food access | 1.05 | 0.84 - 1.31 | 0.658 |
| Medication access | 1.10 | 0.86 - 1.40 | 0.444 |
| Constant | 0.13 | 0.08 - 0.24 | 0.000 |
| *Model 5: Model 4 + coping styles* |  |  |  |
| Problem-focused coping | 1.09 | 0.96 - 1.24 | 0.172 |
| Emotion-focused coping | 1.37 | 1.25 - 1.50 | 0.000 |
| Avoidant coping | 0.89 | 0.80 - 0.99 | 0.030 |
| Supportive coping | 1.27 | 1.17 - 1.39 | 0.000 |
| constant | 0.22 | 0.12 - 0.40 | 0.000 |
